# Retinal drusen counts are increased in inflammatory bowel disease, and with longer disease duration, more complications and associated IgA glomerulonephritis

**DOI:** 10.1038/s41598-022-15232-4

**Published:** 2022-07-11

**Authors:** E. Nicklason, Y. Ham, D. Ng, S. Glance, K. Abel, P. Harraka, H. Mack, D. Colville, J. Savige

**Affiliations:** 1 Department of Medicine, The University of Melbourne, Melbourne Health , Parkville, VIC 3050 Australia; 2 Department of Medicine, The University of Melbourne, Northern Health , Epping, VIC 3076 Australia; 3 Department of Gastroenterology, Northern Health, Epping, VIC 3076 Australia; 4 Department of Ophthalmology, The University of Melbourne, Royal Victorian Eye and Ear Hospital, East Melbourne, VIC 3002 Australia

**Keywords:** Genetics, Immunology, Gastroenterology, Nephrology, Signs and symptoms

## Abstract

Retinal drusen are deposits of inflammatory proteins that are found in macular degeneration and glomerulonephritis and result, in part, from complement activation. This was a cross-sectional observational study of individuals with inflammatory bowel disease (IBD) recruited from a Gastroenterology clinic who underwent non-mydriatic retinal photography. Deidentified images were examined for drusen, and drusen counts and size were compared with matched controls, and examined for clinical associations. The cohort with IBD comprised 19 individuals with ulcerative colitis, 41 with Crohn’s disease and three with indeterminate colitis, including 34 males (54%) and an overall median age of 48 (IQR 23) years. Their median IBD duration was 7 (IQR 10) years, median CRP level was 7 (IQR 14) mg/L, and 28 (44%) had complications (fistula, stricture, bowel resection etc.), while 28 with Crohn’s disease (68%) had colonic involvement. Drusen counts were higher in IBD than controls (12 ± 34, 3 ± 8 respectively, *p =* 0.04). Counts ≥ 10 were also more common (14, 22%, and 4, 6%, *p* = 0.02, OR 4.21, 95%CI 1.30 to 13.63), and associated with longer disease duration (*p* = 0.01, OR 1.06, 95%CI 1.00 to 1.13), an increased likelihood of complications (*p* = 0.003, OR 6.90, 95%CI 1.69 to 28.15) and higher CRP levels at recruitment (*p* = 0.008, OR1.02, 95%CI 1.00 to 1.05). Increased retinal drusen were found in all four individuals with Crohn’s disease and IgA glomerulonephritis. IBD and drusen may share pathogenetic mechanisms and underlying risk factors such as complement activation.

## Introduction

Inflammatory bowel disease (IBD) appears to result from an inappropriate inflammatory response to intestinal microbes in a genetically-susceptible host^1^. It affects at least 1 in 300 individuals^2^, and includes both ulcerative colitis and Crohn’s disease. Ulcerative colitis and Crohn’s disease share some clinical and pathological features but also have important differences^1^. Thus, ulcerative colitis affects mainly the large bowel mucosa and Crohn’s disease includes transmural discontinuous lesions in the terminal ileum; Crohn’s disease is more likely to have complications, such as strictures, fistulae, and perianal disease; both have extra-intestinal manifestations including arthritis, skin features and ocular abnormalities (episcleritis, retinal vasculitis, vasoocclusive disease, choroidal infiltrates, optic neuritis^3^; and Crohn’s disease is more likely to require immunosuppression and biologicals, as well as maintenance therapy to prevent relapses.

Genetic and environmental factors are important in the pathogenesis of both ulcerative colitis and Crohn’s disease. The intestinal microbiota, smoking status^1^ and diet all contribute. Genome-wide association studies (GWAS) have, to date, implicated more than 200 genes or risk alleles^4^ affecting proteins often in the innate (*NOD2*, *TLR4, CARD9, IL23R, STAT3*) and adaptive (*HLA-C, TNFSF15, IRF5, PTPN22*)^5^ arms of intestinal immunity^6,7^. Thirty % of genetic variants are common to both ulcerative colitis and Crohn’s disease, and some are shared with other immune- mediated diseases such as type I diabetes and rheumatoid arthritis^8^. Most of these variants are rare with a small effect size (OR < 1.3).

In the bowel, specialised epithelial cells and the lymphoid tissue are critical in the balance between tolerance of the microbiota and the recognition of pathogens and preventing pathogen invasion. Complement contributes to intestinal immunity through opsonisation of pathogens, the recruitment of inflammatory cells, lysis of bacterial cells by the membrane attack complex, promoting cell death and clearing apoptotic and necrotic cells^9^.

Aberrant complement activity contributes to IBD pathogenesis^10–12^. The normal intestinal epithelium when exposed to bacterial wall lipopolysaccharide produces more C3^13^, which prevents bacterial overgrowth and invasion. Mice deficient in complement proteins do not respond appropriately to intestinal infections^14^. In IBD, the mucous layer overlying the epithelium is defective allowing persistent contact with bacteria and activation of the inflammatory defences, including complement^15^. Components of the alternative complement pathway, in particular, are upregulated in IBD. GWAS have confirmed the role of complement in IBD pathogenesis^16^.

Drusen are retinal deposits that are characteristic of age-related macular degeneration, and are often found where there is alternative complement pathway activation^17^. They comprise cell debris, immune material including complement, and extracellular matrix components^18^.

Drusen also occur in some forms of glomerulonephritis that have an autoimmune or genetic basis such as systemic lupus erythematosus (SLE), IgA glomerulonephritis, C3 glomerulopathy and atypical haemolytic syndrome^19–21^, especially where the alternative complement pathway is affected. IgA glomerulonephritis sometimes complicates IBD too^22^.

Drusen develop from the accumulation of cell debris including cell wall lipid from the highly metabolically-active photoreceptor cells^23^. Complement is activated when the drusen lipid is altered by oxidative stress with increasing age, smoking, poor diet or prolonged ultraviolet light exposure.

There is also a strong genetic predisposition to drusen formation especially with variants in the genes encoding alternate complement pathway components. More than half the susceptibility^24^ in European populations is from variants in the genes for *CFH*^17^ and *ARMS2/HTRA1*^25^*.* CFH is the major inhibitory regulator of the alternative complement pathway, that normally reduces the inflammation from oxidation-modified lipids. The common *CFH* variants lessen complement inactivation, and increase membrane attack complex activity and cell damage^26^. ARMS2/HTRA1 has serine protease activity that weakens the extracellular matrix^27^, allowing retinal deposits to increase in size and promoting local inflammation. It is also directly linked to the complement system and the opsonisation of apoptotic and necrotic cells^28^.

This study examined individuals with IBD for retinal drusen and whether the presence of drusen correlated with any clinical features.

## Methods

### Study design

This was a single centre, cross-sectional observational study of consecutive individuals with IBD recruited from the Gastroenterology clinic of a metropolitan teaching hospital over a 6-month period. Recruitment, data capture, and retinal photography were coordinated in a single episode. Deidentified retinal images were graded for drusen and correlated with clinical features. Results were compared with those from age- and gender- matched hospital controls.

The primary outcome was to determine if drusen were more common in individuals with IBD, and the secondary outcomes to identify if drusen were associated with disease extent, complications or duration. There were no changes to the study design after its commencement and no interim analyses.

This project was approved by the Northern Health Human Research Ethics Committee and written, informed consent was obtained from participants according to the principles of the Declaration of Helsinki. All procedures were performed in accordance with the relevant guidelines and regulations.

### Participants

Consecutive individuals attending a routine clinic review for management of their IBD were invited to participate. They had been diagnosed previously with ulcerative colitis or Crohn’s disease by a specialist gastroenterologist using conventional clinical, radiological, colonoscopic and histopathological criteria^29^. All had been treated according to current international guidelines^30^ and immunosuppression treatment (azathioprine, methotrexate) and the use of biologicals were noted. Disease activity (active or inactive disease) was assessed by the treating clinician and recorded from the clinic charts.

Control participants were age- and gender- matched hospital patients without systemic inflammatory disease who were recruited contemporaneously from general medical and preoperative surgical clinics.

Exclusion criteria were where retinal images were ungradable bilaterally.

### Measurements

#### Clinical features

Participants were assisted to complete a structured questionnaire for demographic details (age, gender), clinical features (diagnosis, large bowel involvement in Crohn’s disease, disease duration), and complications (fistula, stricture, perianal disease, small bowel obstruction, bowel resection, carcinoma, extraintestinal manifestations). Data were confirmed from the participants’ electronic medical records. Laboratory data (C-reactive protein (CRP), serum albumin, estimated glomerular filtration rate (eGFR)) were obtained at recruitment. Drusen risk factors (age, hypertension, smoking history, diabetes) were noted.

#### Retinal imaging and grading

Participants underwent digital retinal imaging centred on the macula and disc of both optic fundi using a non-mydriatic retinal camera (CR5-45, Japan). Deidentified images were examined by two trained graders and a physician independently, using a grid overlay corresponding to the Wisconsin Age-Related Maculopathy Grading System^31,32^. Drusen were counted in the central zones of the inner ring, and in the inferior, superior, temporal and nasal zones of the intermediate and external rings of the grid. Peripheral drusen at least two disc diameters from the foveola were also counted. Numbers from the eye with the greater count were recorded. Drusen ≥ 10 were considered abnormal^31,33^**.** Counts were highly reproducible, with an intra-observer interassay coefficient of variation of 18%.

Drusen were also assessed as small (< 63 µm), medium (63–125 µm) or large (> 125 µm) by comparison with the width of the largest venule where it crossed the ipsilateral disc margin^32^. All images were examined by an ophthalmologist who excluded other causes of the retinal appearance.

### Statistical analyses

This was a pilot study to determine if drusen were more common in IBD than controls and to ascertain all clinical associations so a power calculation was not possible and multiple analyses were undertaken without correction.

Continuous variables were described as mean and standard deviation (for normally distributed data) or median and interquartile range (IQR, where non-normally distributed). Categorical variables were compared using Fisher’s exact test and the one-way ANOVA test. Continuous variables were compared with the student’s t-test or the Mann–Whitney test (non-normally distributed). The relationship of drusen with more extensive disease, complications or disease duration were then calculated using Fisher’s exact test.

Drusen associations were examined with the calculation of odds ratios and 95% confidence intervals with univariate logistic regression analyses. Analyses were performed using the Statistics Package for the Social Sciences (IBM, US). A p-value of less than 0.05 was considered significant.

## Results

### Demographic and clinical features

Sixty-three individuals with IBD were examined, including 19 (30%) with ulcerative colitis, 41 (65%) with Crohn’s disease, and 3 (5%) with indeterminate colitis (Table 1). Five others (7%) had been excluded because of bilaterally ungradable retinal photographs.

**Table 1:** Clinical characteristics of participants with IBD and controls.

Characteristics	Ulcerative colitis (n = 19)	Crohn’s disease (n = 41)	All IBD (n = 63)	Controls (n = 63)	All IBD and Controls OR (95% CI), *p* value
Age (median (IQR), years)	43 (24)	49 (43)	48 (21)	47 (25)	1.00 (0.98 to 1.02), *p =* 0.89
**Sex**					
Female, *n* (%)	9 (47)	19 (46)	29 (47)	29 (46)	1.00 (0.50 to 2.02), *p =* 1.00
Male, *n* (%)	10 (53)	22 (54)	34 (53)	34 (54)	
**Ancestry**					
Northern European, *n* (%)	10 (53)	32 (78)	45 (71)	45 (71)	1.00 (0.46 to 2.17), *p =* 1.00
Southern European, *n* (%)	7 (37)	9 (22)	16 (25)	16 (25)	1.00 (0.45 to 2.23), *p =* 1.00
Asian, *n* (%)	2 (11)	0	2 (3)	2 (3)	1.00 (0.14 to 7.33), *p =* 1.00
**Co-morbidities**					
Hypertension, *n* (%)	3 (16)	7 (17)	11 (17)	24 (38)	**0.33 (0.15 to 0.79), *p =* 0.02**
Smoking history, *n* (%)	9 (47)	25 (61)	8 (13)	39 (62)	0.09 (0.04 to 0.22), *p =* 0.70
Diabetes, *n* (%)	5 (26)	3 (7)	36 (57)	10 (16)	7.07 (3.05 to 16.37), *p =* 0.60
**Laboratory indices**					
CRP (median (IQR), mg/L)	5 (15)	7 (14)	7(14)	10 (46)	*p =* 0.12
Serum albumin (median (IQR), g/L)	36 (7)	37 (6)	37(6)	37 (7)	*p =* 0.84
eGFR (median (IQR), ml/min/1.73 m^2^)	90 (17)	90 (14)	90 (15)	90 (0)	*p =* 0.08
**IBD features**					
Disease duration (median (IQR), years)	8 (11)	7 (12)	7 (10)		
**Anatomical distribution**					
**Ulcerative colitis** (n = 18)					
Proctosigmoiditis, *n* (%)	8 (44)				
Extensive left sided colitis, *n* (%)	4 (22)				
Pancolitis, *n* (%)	6 (33)				
**Crohn’s disease** (n = 41)					
Small bowel only, *n* (%)		13 (32)			
Colonic involvement, *n* (%)		28 (68)			
**Complications** (n = 28)	4 (21)	23 (55)	27 (43%)		
Fistula, *n* (%)	0	2 (5)	2 (3%)		
Stricture, *n* (%)	0	14 (35)	14 (22%)		
Perianal disease, *n* (%)	1 (%)	1 (3)	2 (3%)		
Small bowel obstruction, *n* (%)	0	5 (13)	5 (8%)		
Bowel resection, *n* (%)	2 (6)	11 (28)	13 (21%)		
Adenomas, *n* (%)	0	2 (5)	2 (3%)		
Bowel cancer, *n* (%)	0	1 (3)	1 (2%)		
Extraintestinal manifestations, *n* (%)	5 (28)	8 (20)	13 (21%)		

All IBD included participants with UC, CD or indeterminate disease; Anatomical distribution was not known in one individual with ulcerative colitis and five with Crohn’s disease. N/A not applicable.

Significant values are in bold.

The final cohort comprised 34 men (54%) and 29 women (46%) with a median age of 48 years (IQR 37–60). They were of Northern European (45, 71%), Southern European (16, 25%) or Asian (2, 3%) ancestry.

Eleven (17%) had treated hypertension, 8 (13%) were current or former smokers, and 36 (57%) had diabetes. They had a median IBD duration of 7 years (IQR 10), 28 (44%) had complications, and 28 with Crohn’s disease (68%) had colonic involvement. Their median CRP was 7 (IQR 14) mg/L and their median serum albumin was 37 (IQR 6) g/L with a median eGFR of 90 (IQR 15) ml/min/1.73m^2^.

Participants with IBD were not different from controls in age, sex, ethnicity or comorbidities except that they were less likely to have hypertension (*p* = 0.02, OR 0.33, 95%CI 0.15 to 0.79) (Table 1).

### Ophthalmic examinations

None of the participants with IBD had any of the characteristic retinal features. Coincidental abnormalities included a choroidal naevus, macular scar, thrombosed vessel, and glaucomatous optic disc. Retinal haemorrhages were present in 5 individuals with IBD (8%) and 5 controls (8%), and exudates were found in 7 with IBD (11%) and 8 controls (12%).

### Drusen

**Figure 1:**
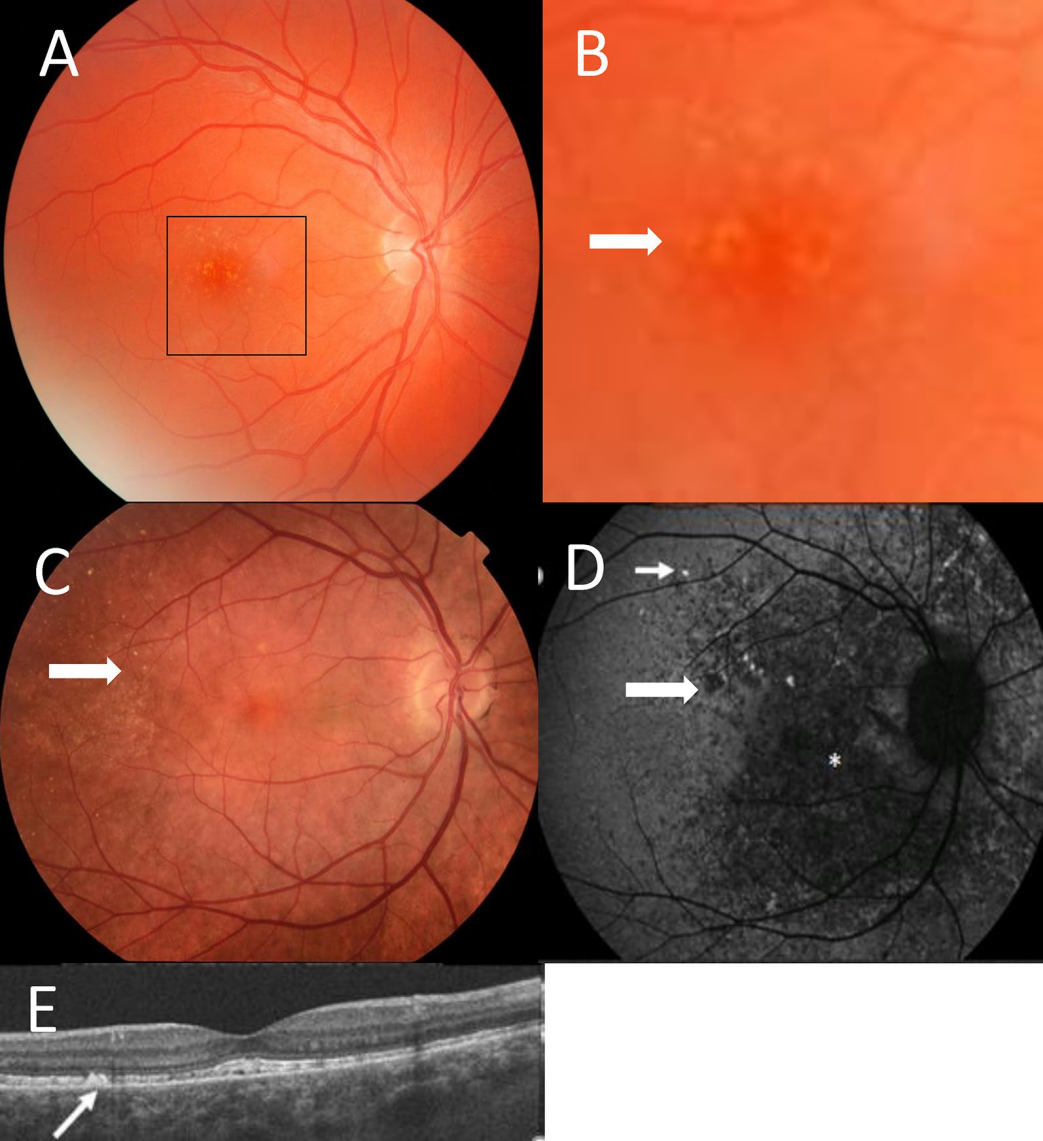
(**A**) Right optic fundus from an individual with Crohn’s disease demonstrating clusters of small central drusen; (**B**) enlarged view of box in A demonstrating drusen more clearly (arrow); (**C**). right optic fundus from an individual with ulcerative colitis demonstrating drusen (arrow); (**D**). autofluorescence view of the fundus in (**C**). demonstrating hyperfluorescent drusen (arrow), hypofluorescent drusen (arrow) and areas of hypofluorescence (asterisks); and (**E**). focal drusen in (**C**) and (**D**) resulting in irregularity of outer retinal layers on optical coherence tomography (OCT, Heidelberg Engineering) (arrow).

Participants with IBD had a mean drusen count of 12 ± 34 compared with 3 ± 8 in hospital controls (*p =* 0.04) (Table 2). Abnormal counts ≥ 10 were more common in IBD than controls (*p =* 0.02, OR = 4.21 (1.30 to 13.63). Drusen in IBD were more widely distributed than in controls (*p =* 0.01, OR 3.31, 95%CI 1.12 to 9.85) but were not larger. Drusen were mainly small, soft, and well-circumscribed (Fig. 1). Optical coherence tomography (OCT) demonstrated that they were located beneath the retinal pigment epithelium in the same site as the drusen found in SLE, IgA glomerulonephritis and dense deposit disease (Fig. 1)^19–21^.

**Table 2:** Drusen counts, location and size in all IBD, controls, and ulcerative colitis or Crohn’s disease.

Drusen	All IBD (n = 63)	Hospital controls (n = 63)	All IBD and Controls OR (95% CI), p value	Ulcerative colitis (n = 19)	Crohn’s disease (n = 41)	Ulcerative colitis and Crohn’s disease, OR (95%CI) p value
Total drusen counts (mean, SD)	12 ± 34	3 ± 8	***p =* 0.04**	4 ± 6	16 ± 42	*p =* 0.22
≥ 10 drusen (n, %)	14 (22%)	4 (6%)	**4.21 (1.30 to 13.63), *p =* 0.02**	3 (16%)	10 (24%)	0.58 (0.14 to 2.42) *p =* 0.46
Drusen in ≥ 4 macular quadrants (n, %)	14 (22%)	5 (8%)	**3.31 (1.12 to 9.85), *p =* 0.01**	4 (21%)	9 (22%)	0.95 (0.25 to 3.58) *p =* 0.94
Any medium or large drusen (n, %)	7 (11%)	6 (10%)	1.19 (0.38 to 3.75), *p =* 1.00	4 (21%)	3 (7%)	3.38 (0.67 to 16.93) *p =* 0.14

Significant values are in bold.

### Clinical associations

Participants with ulcerative colitis had a mean drusen count of 4 ± 6 (n = 19) compared with Crohn’s disease where the mean count was 16 ± 42 (n = 41) (*p =* 0.22). However counts ≥ 10, distribution and size were not different in ulcerative colitis and Crohn’s disease.

Participants with IBD and disease duration ≥ 10 years had a mean drusen count of 25 ± 53 (n = 24) and those with a duration < 10 years had a mean count of 2 ± 4 (n = 35) (*p =* 0.04).

Participants with IBD and complications had a mean drusen count of 22 ± 49 (n = 28) compared with 2 ± 3 in those with uncomplicated disease (n = 31) (*p =* 0.02).

Participants with Crohn’s disease and large bowel involvement had a mean drusen count of 22 ± 50 (n = 28) compared with 2 ± 4 in those with disease limited to the small bowel (n = 13) (*p =* 0.16).

The highest drusen counts (124, 122 and 108) were found in the periphery (Fig. 1). These occurred in three individuals with Crohn’s disease aged 39, 56 or 79 years, and all with disease duration of at least 10 years. All three had colonic involvement, and one had complications with a small bowel obstruction. None had any features of extraarticular Crohn’s disease.

### Clinical associations of drusen counts ≥ 10

Fourteen individuals with IBD (22%) and 4 controls (6%) had drusen ≥ 10 (*p =* 0.02, OR 4.21, 95% CI 1.30 to 13.63). The participants with IBD included 3 with ulcerative colitis, 10 with Crohn’s disease and one with indeterminate colitis.

Drusen counts ≥ 10 were not more likely with Crohn’s disease than ulcerative colitis (*p =* 0.35, OR 0.55, 95%CI 0.05 to 6.60) (Table 3).

**Table 3:** Central drusen counts ≥ 10 and clinical associations in all participants with IBD.

**Clinical features**	** ≥ 10 drusen** (n = 14)	** < 10 drusen** (n = 49)	**OR (95% CI), p value**
**Age **(median (IQR), years)	55 (22)	45 (25)	**1.02 (0.98 to 1.07), *p =* 0.04**
**Sex**			
Female	7 (50%)	22 (45%)	1.18 (0.36 to 3.90), *p =* 0.60
Male	7 (50%)	26 (53%)	
**Ethnicity** (n, %)			
Northern European	10 (71%)	35 (71%)	1.00 (0.27 to 3.72), *p =* 0.61
Southern European plus Asian	4 (28%)	14 (29%)	
**Co-morbidities**			
Hypertension	1 (7%)	9 (18%)	0.342 (0.04 to 2.96), *p =* 0.28
Smoking history	4 (29%)	32 (65%)	**0.213 (0.06 to 0.78), *p =* 0.01**
Diabetes	3 (21%)	5 (10%)	2.40 (0.50 to 11.61), *p =* 0.25
**Disease type** (n, %)			
Ulcerative colitis	3 (21%)	16 (33%)	0.55 (0.05 to 6.60), *p =* 0.35
Crohn’s disease	10 (71%)	31 (63%)	
**Disease extent in Crohn’s disease**			
Colonic involvement (n = 28)	9 (32%)	19 (68%)	5.68 (0.64 to 50.72), *p =* 0.13
No colonic involvement (n = 13)	1 (8%)	12 (92%)	
**Active disease**	10 (77%)	39 (85%)	0.60 (0.13 to 2.74), *p =* 0.68
**Immunosuppression (Aza, MTX)**	7 (54%)	23 (49%)	1.22 (0.36 to 4.17), *p =* 1.00
**Disease duration** (median (IQR) years)	11 (14)	5 (9)	**1.06 (1.00 to 1.13), *p =* 0.01**
**Complications **(n, %)	11 (79%)	17 (35%)	**6.90 (1.69 to 28.15), *p =* 0.003**
Extra-intestinal manifestations (n = 13)	5 (36%)	8 (16%)	2.85 (0.75 to 10.77), *p =* 0.11
**Laboratory indices**			
CRP (median (IQR), mg/L)	14 (94)	5 (13)	**1.02 (1.00 to 1.05), *p =* 0.008**
Serum albumin (median (IQR), g/L)	36 (5)	37 (6)	0.93 (0.77 to 1.13), *p =* 0.36
eGFR (median (IQR), ml/min/1.73m^2^)	89 (20)	90 (14)	1.00 (0.96 to 1.06), *p =* 0.47

Aza, MTX – azathioprine, methotrexate.

Significant values are in bold.

Participants with IBD and drusen ≥ 10 were older (*p =* 0.04, OR1.02, 95%CI 0.98 to 1.07) and less likely to be current or former smokers than those with < 10  (*p =* 0.01, OR 0.21, 95%CI 0.06 to 0.78) (Table 3). Drusen counts ≥ 10 were not associated with gender, ancestry, or the diagnosis of hypertension or diabetes (p all NS).

However drusen counts ≥ 10 were more common in individuals with IBD of longer disease duration (*p =* 0.01, OR 1.06, 95%CI 1.00 to 1.013), increased complications (*p =* 0.003, OR 6.90, 95%CI 1.69 to 28.15) or with higher CRP levels at recruitment (*p =* 0.008, OR1.02, 95%CI 1.00 to 1.05). Drusen counts ≥ 10 were not more common in patients assessed to have active disease clinically (*p =* 0.68, OR 0.60, 95% CI 0.13 to 2.74) nor were they affected by current immunosuppressive treatment (*p =* 1.00, OR 1.22, 95%CI 0.36 to 4.17). They were also not associated with extraintestinal manifestations (*p =* 0.11, OR2.85, 95%CI 0.75 to 10.77), reduced serum albumin (*p =* 0.36, OR 0.93, 95%CI 0.77 to 1.13) or lower eGFR levels (*p =* 0.47, OR 1.00, 95%CI 0.96 to 1.06).

### Association with IgA glomerulonephritis

In addition, four individuals with Crohn’s disease had renal-biopsy-proven IgA glomerulonephritis. All had increased drusen counts of 22, 40, 49 or 222. These drusen occurred in two men and two women, aged 39, 45, 56 or 79 years, with disease of at least 10 years’ disease.

## Discussion

Retinal drusen are more common in IBD than other hospital patients. Abnormal drusen counts were found in about 20% of individuals with IBD, and were associated with longer disease duration, more frequent complications and higher CRP levels at a clinic visit. These associations suggest that drusen were more abundant with more prolonged and possibly more active disease. However abnormal drusen counts did not correlate with clinical assessments of disease activity nor were counts lower with immunosuppression. Drusen were also increased in all individuals with IgA glomerulonephritis complicating Crohn’s disease.

The association of drusen with IBD suggests shared risk factors and pathogenetic mechanisms. Drusen in macular degeneration have both genetic and environmental risk factors. The major environmental risks are age, smoking, hypertension, ancestry, diabetes and renal impairment^34^. Most participants with IBD in this study were too young for the drusen to be age-related, and drusen were not associated with smoking despite it also being a risk factor for Crohn’s disease^35^. In addition, drusen were not associated with hypertension, ancestry, diabetes or renal impairment.

Genetic risk factors for drusen in macular degeneration affect pathways involved in complement activation, fatty acid oxidation, membrane integrity and apoptosis ^23,36^. Some of the genetic risk factors for macular degeneration are also risk factors for IBD such as alleles in the complement pathway genes, and sometimes, while there is no association with a risk allele, the corresponding protein has been implicated (VEGFA, ABCA1)^37,38^. The main difference in genetic risk factors is the lack of immunological processes in macular degeneration.

The major risk allele for drusen in macular degeneration affects the alternative complement pathway regulator, CFH. Complement activation and genetic risk alleles in complement pathway genes occur in both drusen and IBD development^23,36^**.** Drusen incorporate photoreceptor cell membrane lipid that is oxidised by smoking and sunlight, and activates complement, inducing inflammation and apoptosis. In IBD, exposure of microbial cell wall lipid to normal intestinal epithelium increases C3 production^13^. The defective intestinal mucosa in IBD allows ongoing bacterial contact and further activation of the alternative complement pathway^15,16^. CRP also binds bowel wall bacteria and triggers classical pathway complement activation^39^.

Complement involvement is different in Crohn’s disease and ulcerative colitis, and increased complement activation in Crohn’s disease may explain the greater drusen number. In Crohn’s disease the intestinal mucosa produces more C3 and C4^10^, and crypt abscess formation induces further C3 expression^13,14,40^. In addition, more extensive Crohn’s disease may be associated with increased complement activation since colonic epithelial cells express more C3 than ileal cells^13^ and the major C3 stimulator, Toll-like receptor 4 (TLR-4) is up-regulated in the colon in mouse models of IBD^13^.

Drusen were present in all individuals with Crohn’s disease and biopsy proven IgA glomerulonephritis in this study. IgA glomerulonephritis has been reported previously in both ulcerative colitis and Crohn’s disease^41,42^ and was excluded in other study participants based on their normal urinary sediments. Drusen have been described previously in only occasional cases of IgA glomerulonephritis^21,43^ but drusen and glomerular immune deposits in other forms of glomerulonephritis share their composition of complement, immunoglobulins, glycoproteins, and extracellular matrix^44,45^. Complement activation and complement risk alleles are important in both the pathogenesis of macular degeneration and Crohn’s disease, but also IgA glomerulonephritis.

Overall the drusen found in IBD appeared to be smaller than those found in macular degeneration and dense deposit disease but of similar size to those in SLE^20^ and IgA glomerulonephritis^21^. Drusen were located mainly in the central retina, in the same location as for IgA glomerulonephritis, SLE and dense deposit disease ^19–21^.

Drusen were not present in all individuals with IBD or Crohn’s disease who were examined. Retinal imaging is relatively insensitive for drusen detection^20^ and smaller drusen may be even more abundant than found here. This means that drusen large enough to be visualised on retinal imaging are associated with longer disease duration, more complicated disease and higher CRP levels at a clinic visit. There was no correlation with clinically-assessed active disease nor fewer drusen with immunosuppression. Importantly, the drusen in IBD, unlike those in macular degeneration, do not affect vision.

The drusen in IBD are unlikely to be coincidental because counts were higher than in other hospital patients, and drusen were present in patients who also had IgA glomerulonephritis. Drusen or a drusen-like appearance secondary to other retinal disease was excluded by independent examination of all images by an ophthalmologist.

The strengths of this study were the clinical characterisation of study participants, and the standardisation and reproducibility of drusen counts. The study’s major limitations were the relatively small cohort and the lack of a more objective assessment of disease activity such as a Disease Activity or Harvey-Bradshaw index. Further studies are required to confirm the clinical associations of drusen in IBD, and the association of drusen with Crohn’s disease, especially with colonic involvement.

Retinal drusen are more common in IBD, and associated with longer disease duration, more complicated disease and higher CRP levels at a clinic visit. The identification of drusen in IBD suggest that complement activation is important in IBD pathogenesis. Treatments used for drusen including those targeting the complement pathway in macular degeneration may be relevant in IBD. The finding of drusen in both IBD and IgA glomerulonephritis further suggests a role for complement activation in these conditions.

## Data Availability

The datasets generated and/or analysed during the current study are not publicly available because the authors hope to undertake further analysis, but are available from the corresponding author on reasonable request.
